# Involvement of 5mC DNA demethylation *via* 5-aza-2'-deoxycytidine in regulating gene expression during early somatic embryo development in white spruce (*Picea glauca)*

**DOI:** 10.48130/fr-0023-0030

**Published:** 2023-12-26

**Authors:** Ying Gao, Xiaoyi Chen, Chengbi Liu, Huanhuan Zhao, Fengbin Dai, Jian Zhao, Jinfeng Zhang, Lisheng Kong

**Affiliations:** 1 Tea Research Institute, Shandong Academy of Agricultural Sciences, Jinan 250100, China; 2 State Key Laboratory of Efficient Production of Forest Resources, National Engineering Research Center of Tree Breeding and Ecological Restoration, Key Laboratory of Genetics and Breeding in Forest Trees and Ornamental Plants, Ministry of Education, The Tree and Ornamental Plant Breeding and Biotechnology Laboratory of National Forestry and Grassland Administration, College of Biological Sciences and Biotechnology, Beijing Forestry University, Beijing 100083, China; 3 Department of Biology, Centre for Forest Biology, University of Victoria, Victoria, BC V8P 5C2, Canada; 4 Zoucheng Improved Variety Experiment and Extension Center, Zoucheng 273518, China

**Keywords:** 5-aza-dC, Pre-maturation treatment, *MSH7*, *JMJ14*, *CalS10*, Methylated regions

## Abstract

DNA methylation plays a crucial role in the development of somatic embryos (SEs) through the regulation of gene expression. To examine the impact of DNA methylation on gene expression during early SE development in *Picea glauca*, the demethylation reagent 5-aza-dC (5-aza-2′-deoxycytidine) was employed to modify DNA methylation regions and levels during the pre-maturation stage of somatic embryogenesis. The application of 2.0 µM 5-aza-dC did not induce toxicity to SEs in early development. Following treatment, the global DNA methylation level decreased significantly on the 7^th^ day of pre-maturation and the 1^st^ week of maturation. Methylated DNA immunoprecipitation (MeDIP) sequencing revealed that differentially methylated regions, as analyzed through Gene Ontology (GO), were related to plant development and reproduction and that they were hypomethylated on the 3^rd^ day but hypermethylated on the 7^th^ day in 5-aza-dC-treated embryogenic tissues. These findings indicate that 5-aza-dC treatment positively impacts early SE development, which was inhibited following 7 d of treatment. The expression of *MSH7*, *JMJ14*, and *CalS10* was associated with DNA methylation, epigenetic regulation, and somatic embryogenesis. Further analysis of methylated regions revealed that the expression profiles of *MSH7*, *JMJ14*, and *CalS10* were correlated with altered DNA methylation, suggesting DNA methylation at 5 mC may play a role in controlling the expression of these genes and regulating the early development of SEs in *P. glauca*. This study offers new insights into the regulation of somatic embryogenesis in conifers.

## Introduction

Haberlandt first introduced the concept of plant cell totipotency in 1902. Subsequently, Muir et al. demonstrated that single cells from *Tagetes erecta* can divide to generate small cell clusters^[1]^. In 1958, somatic embryos (SEs) were induced in carrot, which ultimately developed into whole plants^[2]^.

Somatic embryogenesis involves the dedifferentiation of somatic cells, which is triggered by specific conditions, allowing them to regain totipotency and pluripotenency for regeneration from single cells. This process bears similarity to the development of zygotic embryos^[3]^. Somatic embryogenesis is particularly advantageous for tree breeding, especially in plant species with prolonged growth cycles and challenges in obtaining mature seeds. Additionally, it holds relevance in genetic research^[4,5]^. Despite the establishment of somatic embryogenesis systems for spruces since 1985^[6]^, the molecular mechanism underlying how somatic cells initiate reprogramming to regain the ability of cell division and develop into whole plants remains unclear.

Studies on various plants have revealed a close association between somatic embryogenesis and epigenetic factors^[7,8]^. Epigenetic regulatory mechanisms encompass DNA methylation, chromatin remodeling, histone modification, and non-coding RNAs (ncRNA)^[9]^. Among these, DNA methylation is a well-conserved and widely-recognized epigenetic mechanism, typically occurring at the fifth carbon of the cytosine base in CpG islands through DNA methyltransferase^[10,11]^. DNA methylation at 5 mC plays important roles in seed development^[12,13]^, vegetative growth and pattern formation^[14]^, fruit ripening^[15,16]^, and environmental stimulus response^[17,18]^ throughout the plant's life cycle.

Recent investigations have demonstrated the involvement of DNA methylation in the development of SEs in higher plants^[19−21]^. For example, in cotton, CHH hypomethylation has been identified as a distinct epigenetic mark for embryogenic re-differentiation in highly embryogenic genotypes, with genotype-dependent methylation modes^[19]^. Similarly, during the early stages of longan somatic embryogenesis, hypomethylation was predominantly observed in CHH contexts^[22]^. In sweet orange, somaclonal variation analysis revealed a retrotransposon insertion as well as DNA modification alternations in the CLAVATA3/EMBRYO SURROUNDING REGION-RELATED 16 gene, potentially linking it to somatic embryogenesis. In *Norway spruce*, embryogenic cells exhibited lower levels of CG and CHG methylation but higher levels of CHH methylation compared to needle cells^[23]^. Thus, DNA methylation reprogramming is evident in both angiosperms and gymnosperms during somatic embryogenesis.

Few studies have addressed the epigenetic regulatory mechanisms of somatic embryogenesis, and there is an absence of reports on the regulatory role of DNA methylation during the early stage of SE development in conifers. Therefore, the aims of this study were to determine and analyze the differences in DNA methylation levels of the key genes during early SE developmental stage. To answer the question, the demethylation reagent 5-aza-dC (5-aza-2′-deoxycytidine) was used to investigate the significance of DNA methylation in somatic embryogenesis. The study sought to establish correlations between gene expression profiles and DNA methylation modifications in both genes and downstream regions, thus aiming to identify genes implicated in the development of SEs.

## Materials and methods

Embryogenic tissue was induced from mature zygotic embryos of *P. glauca* and tested for embryogenic capability^[24]^. From various genotypes, WSP3, a cell line of high embryogenic capability was selected and used for the experiments in this study. The SE development process mainly includes cultures of maintenance, suspension, pre-maturation, and maturation. All cultures were maintained in the dark at 23 ± 1 °C. During the maintenance stage, embryogenic tissues were sub-cultured once two weeks with half-strength modified Litvay's medium (1/2 mLV) supplemented with 1 mg/L 2,4-D, 0.5 mg/L 6-BA, 0.5 g/L hydrolyzed casein, 0.5 g/L glutamine, 10 g/L sucrose, and 3 g/L gellan gum^[25]^. For suspension culture, the embryogenic tissues were transferred to liquid maintenance medium without gellan gum in flasks and cultivated for 2 weeks in a shaker spinning at 100 rpm. The embryogenic tissues were collected by vacuum filtration, and 0.8 g of the collected tissues was added into 50 ml of liquid pre-maturation medium (1/2 mLV basal medium, 30 µM ABA, 0.4 g/L hydrolyzed casein, 0.5 g/L glutamine, 10 g/L sucrose) in a 150-ml flask and cultivated for 1 week in a shaker spinning at 100 rpm. Finally, the embryogenic tissues were transferred into maturation medium (1/2 mLV basal medium, 45 µM ABA, 0.2 g/L hydrolyzed casein, 0.4 g/L glutamine, 30 g/L sucrose, 10 g/L maltose, 6 g/L gellan gum) and cultured for 6 weeks in the dark. All culture media were adjusted to pH 5.8 before sterilization.

### 5-aza-dC treatment and SE maturation

To select the optimal concentration, 5-aza-dC was dissolved with dimethylsulfoxide (DMSO) to prepare a stock solution of 62.5 g/L and then added into pre-maturation medium to prepare working solutions of 0.5, 1.0, 1.5, 2.0, 2.5, and 5.0 μM. As a negative control, 10.944 μL DMSO was added into the suspension culture. After treatment with 5-aza-dC, the embryogenic tissues were sampled to evaluate viability. The experiment was conducted in triplicate, with 1.0 g of embryogenic tissue and 50 ml of pre-maturation medium used for each treatment.

### Viability of SEs

To assess the viability, the embryogenic tissues were stained with 0.8% (w/v) 2,3,5-triphenyltetrazolium chloride (TTC). First, TTC was dissolved in a 2:1 solution of 0.05 M sodium phosphate buffer (pH 7.5) and the pre-maturation medium for staining as described by Towill & Mazur^[26]^. After staining for 18 h, the embryogenic tissues were rinsed once with distilled water and then soaked in 95% (v/v) ethanol for 30 min to extract the red formazan. The absorbance of the extracted solution was detected at 485 nm. The stained embryogenic tissues were observed with an M205FA Leica stereo-fluorescence microscope, and the images were processed with LAS X software. The viability assay was repeated three times, and the mean viability was calculated based on three biological replicates.

### RNA extraction

Total RNA was extracted using an RNAprep Pure Plant Kit (Tiangen Biotech Co., Ltd., Beijing, China) according to the manufacturer's instructions. RNA purity and concentration were analyzed with a Nanodrop 8000 Spectrophotometer (Thermo Fisher Scientific, Waltham, MA, USA). RNA integrity was assessed with a Bioanalyzer 2100 System (Agilent Technologies, Santa Clara, CA, USA) using an RNA Nano6000 Assay Kit.

### Detection of global DNA methylation

To evaluate the status of DNA methylation, DNA was extracted using the CTAB method^[27]^. DNA was purified and hydrolyzed according to the protocol by Gao et al.^[28]^, and DNA methylation was detected with a Waters E2695 HPLC System (Milford, MA, USA) and a Phenomenex C18 column (cat. no. 00G-4252-E0, 250 mm × 4.6 mm, 5 µm). The DNA methylation level was calculated as (5 mC/(5 mC + C)) × 100%.

### RNA-seq of embryogenic tissue

RNA high throughput sequencing was performed by Cloud-Seq Biotech (Shanghai, China). rRNA was removed from total RNA using a NEBNext rRNA Depletion Kit (New England Biolabs, Inc., Ipswich, MA, USA), and RNA libraries were constructed using a NEBNext Ultra II Directional RNA Library Prep Kit (New England Biolabs, USA), according to the manufacturer's instructions. Libraries were quality-controlled and quantified using the BioAnalyzer 2100 System (Agilent Technologies, Inc.). Library sequencing was performed on an Illumina Novaseq System with 150-bp paired end reads.

Paired-end reads were obtained from an Illumina Novaseq 6000 System and quality-controlled by Q30. After 3ʹ adaptor trimming and low-quality read removal by cutadapt software (v1.9.3), the high-quality clean reads were aligned to the reference genome (Pabies-1.0)^[29]^ with hisat2 software (v2.0.4). HTSeq software (v0.9.1) was used to obtain the raw count, whereas edgeR was used to perform the normalization, and then differentially expressed genes were identified by *p*-value ≤ 0.05 and |fold change| ≥ 2.0. Gene Ontology (GO) pathway enrichment analysis was performed based on the differentially expressed mRNAs.

### Transcriptomic co-expression network analysis

To divide unigenes with |(fold change)| > 5 into different modules with similar expression spectra, the WGCNA R package (ver. 1.70-3) was used to construct the weighted gene co-expression network^[30,31]^. First, the adjacency matrix was constructed *via* the power formula (adjacency = |(1+ Pearson correlation)/2|^β^). After calculating the soft threshold parameter β, the adjacency matrix was transformed into a topological overlap matrix, and the corresponding dissimilarity (1-TOM) was determined. The number of genes in the module was set to at least 30, and hierarchical clustering was performed on the base of the TOM measurement method. All the network maps were drawn by Cytoscape software (ver. 3.8.2), which was based on the correlation analysis.

### MeDIP of embryogenic tissue

To extract DNA, a Super Plant Genomic DNA (Polysaccharides & Polyphenolics-rich) Kit (Tiangen Biotech Co., Ltd.) was used, according to the manufacturer's instructions. DNA purity and concentration were checked with a Nanodrop 8000 Spectrophotometer.

High throughput sequencing was performed by CloudSeq Biotech Inc. Genomic DNA was precipitated with ethanol and sonicated to 100–500 bp using a Bioruptor (Diagenode). Sonicated DNA was end repaired, A-tailed, and ligated to adapters using a NEBNext Ultra DNA Library Prep Kit (NEB). MeDIP was performed with a monoclonal antibody against 5-methylcytosine (Active Motif, Carlsbad, CA, USA), according to the manufacturer's protocol. MeDIP DNA libraries were quantified using a Quant-iT PicoGreen dsDNA Kit (Thermo Fisher Scientific) and subjected to high-throughput 150 bp-end sequencing on a Illumina HiSeq4000 System, according to the manufacturer's instructions.

After sequencing, Q30 was used to perform quality control. After 3ʹ adaptor trimming and low-quality read removal by cutadapt software, high quality clean reads were generated. These clean reads were aligned to reference genome (*P. abies*) using bowtie2 software (v2.2.4) with default parameters. Peak calling was performed with MACS software (v1.4.3). Differentially methylated regions were identified by diffReps software (v1.55.4). The methylated regions were then annotated with the gtf file to connect the peak information with the gene annotation. The enriched methylated peaks were visualized in UCSC Genome Browser.

### Quantitative real-time PCR analysis

To validate the results of transcriptome analysis, qRT-PCR was employed to determine the expression of hubgenes (Supplemental Table S1) using an Applied Biosystems QuantStudio 6 Real-Time PCR System (ABI, Carlsbad, CA, USA) and a Hieff UNICON Universal Blue SYBR Green Master Mix (Yeason Biotechnology Co., Ltd., Shanghai, China) as previously reported^[32]^. PCR primers were designed using primer-blast (www.ncbi.nlm.nih.gov/tools/primer-blast/) with default parameters, except the product size (100–250 bp), and the primers were synthesized by Tsingke Biotech Co., Ltd. (Beijing, China). The PCR cycling conditions were as follows: 40 cycles at 95 °C for 30 s, 95 °C for 3 s, and 60 °C for 20 s. Reaction specificity was ensured by analyzing the melting curves. The relative expression levels of the different genes were calculated by the 2^−ΔΔCᴛ^ method using the *EF1* gene as the control^[33]^. qRT-PCR analysis was performed in triplicate.

### Statistical analysis

All experiments were performed at least three independent times (N ≥ 3). Duncan's multiple range test was carried out using SPSS software (IBM, Armonk, NY, USA), with the level of significance set at *p* < 0.05.

## Results

### Effects of 5-aza-dC treatment on the viability of embryogenic tissues

**Figure 1:**
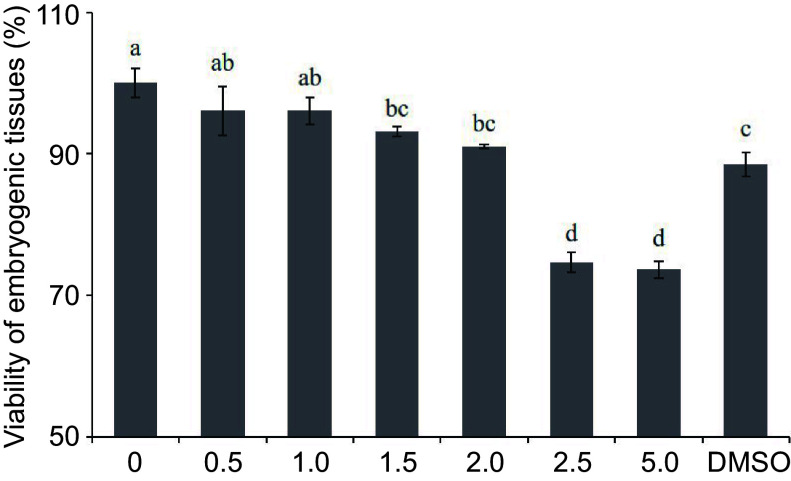
Effect of 5-aza-dC treatment on the viability of embryogenic tissue by the TTC assay. The embryogenic tissues were treated with 0, 0.5, 1.0, 1.5, 2.0, 2.5, and 5.0 µM 5-aza-dC or 5.0 µM DMSO.

**Figure 2:**
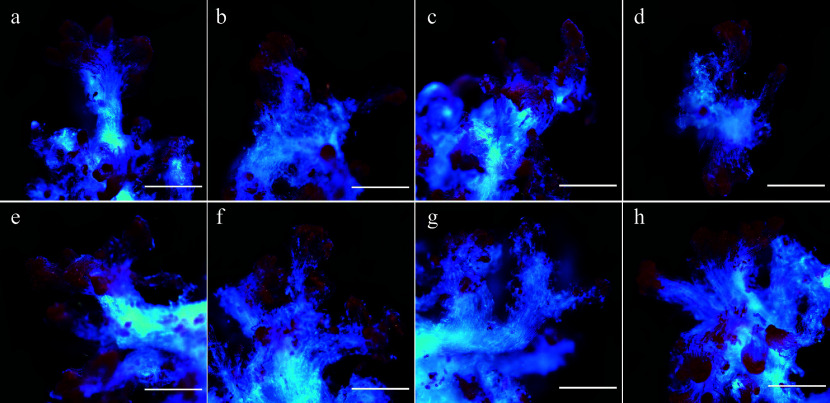
Microscopic observation of 5-aza-dC treated embryogenic tissues by TTC staining. The embryogenic tissues in (a)–(g) were treated with 0, 0.5, 1.0, 1.5, 2.0, 2.5, and 5.0 µM 5-aza-dC, and the embryogenic tissues in (h) were treated with 5.0 µM DMSO (negative control). All bars = 2.2 mm.

To determine the optimal concentration of 5-aza-dC, we conducted a TTC assay to assess the viability of embryogenic tissues and observe the developmental conditions of SEs at various 5-aza-dC concentrations. As shown in Figs 1 & 2, there was no significant difference in tissue viability among treatments with 0, 0.5, and 1.0 µM 5-aza-dC, all of which exceeded a viability rate of 96%. The tissue viability of treatments with 1.5 and 2.0 µM 5-aza-dC was above 90%, similar to that of treatments with 0.5 and 1.0 µM 5-aza-dC. The tissue viability of the negative treatment group (DMSO) was 88%, close to that of treatments with 1.5 and 2.0 µM 5-aza-dC. However, the viability of embryogenic tissues exposed to 2.5 and 5.0 µM 5-aza-dC was notably lower than the others and the development of SEs was poor. These results suggest that there is no significant harm to embryogenic tissues when the 5-aza-dC concentration is equal to or less than 2.0 µM, while embryogenic tissues are seriously harmed when the concentration is equal to or more than 2.5 µM. Therefore, a concentration of 2.0 µM is deemed the most suitable for exploring the function of DNA methylation during SE development.

### Effects of 5-aza-dC on SE development

**Figure 3:**
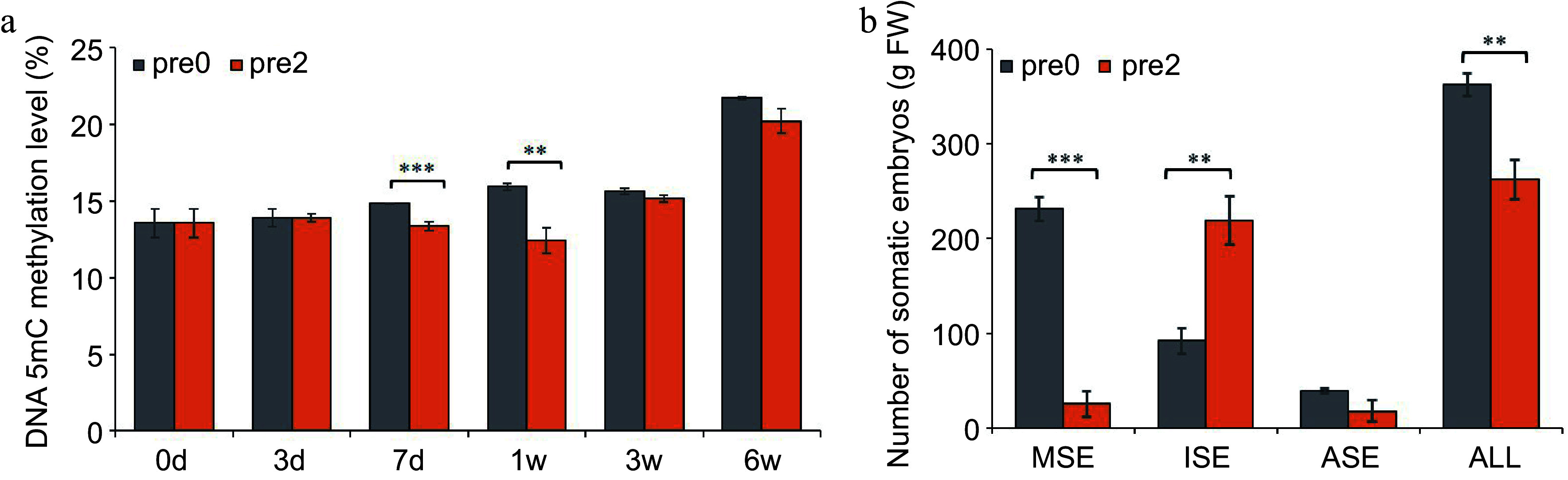
Effect of 5-aza-dC on the development of SEs. (a) The DNA methylation levels of embryogenic tissues on the 0, 3^rd^, 7^th^ d of pre-maturation treatment and the 1^st^, 3^rd^, 6^th^ week of maturation culture. (b) The numbers of mature somatic embryos (MSE), immature somatic embryos (ISE), abnormal somatic embryos (ASE), and total somatic embryos (ALL). Pre0 and pre2 represent untreated and 5-aza-dC treated embryogenic tissues, respectively.

To provide an understanding of DNA methylation dynamics and analyze the impact of 5-aza-dC treatment on somatic embryogenesis, we measured global DNA methylation and embryogenic capability. As shown in Fig. 3a, the DNA methylation level of untreated embryogenic tissues gradually increased from 13.6% to 21.7% during SE development, whereas the DNA methylation level of embryogenic tissues treated with 2.0 µM 5-aza-dC slightly increased, reaching only 20.2%. On the 7^th^ day of pre-maturation and the 1^st^ week of maturation, the DNA methylation level of 5-aza-dC treated embryogenic tissues was significantly lower than that of untreated embryogenic tissues, suggesting that 5-aza-dC treatment leads to lower methylation levels during the early somatic embryogenesis.

**Figure 4:**
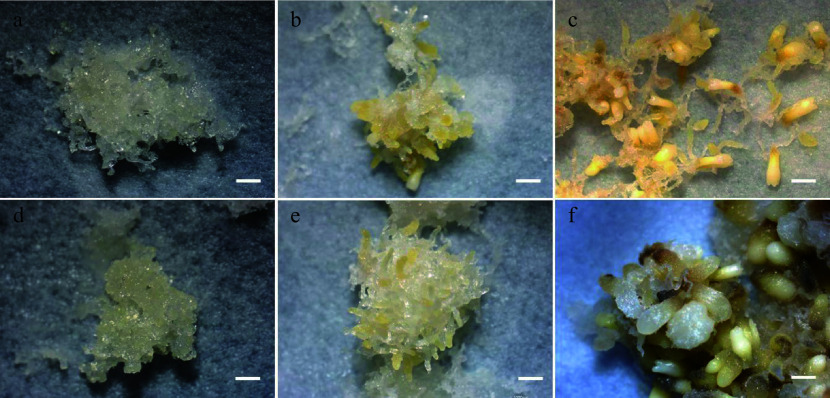
SE development in maturation cultures. (a) Untreated and (d) 5-aza-dC treated embryogenic tissues on the 1^st^ week of maturation culture. (b) Untreated and (e) 5-aza-dC treated embryogenic tissues on the 3^rd^ week of maturation culture. (c) Untreated and (f) 5-aza-dC treated embryogenic tissues on the 6^th^ week of maturation culture. Bar = 1,000 µm.

The morphology of SEs was observed at the 1^st^, 3^rd^, and 6^th^ week during SE maturation culture (Fig. 4). A substantial number of early stage SEs was observed in embryogenic tissues treated with 5-aza-dC (Fig. 4e) compared with untreated embryogenic tissues (Fig. 4b). Furthermore, the number of immature SEs in 5-aza-dC treated embryogenic tissues (Fig. 4f) was significantly higher than that in untreated embryogenic tissues (Fig. 4c). By the 6^th^ week of maturation, the total number of SEs, including mature, immature, and abnormal SEs, was counted (Fig. 3b). In untreated embryogenic tissues, the total number of SEs was 363/g FW, comprising 231/g FW of mature SEs, 92/g FW of immature SEs, and 40/g FW of abnormal SEs. Conversely, in 5-aza-dC treated embryogenic tissues, the number of mature SEs was only 26/g FW, while the number of immature SEs was 219/g FW, indicating that the lower methylation levels can inhibit the development of SEs.

### MeDIP sequencing and GO analysis of methylated regions

**Figure 5:**
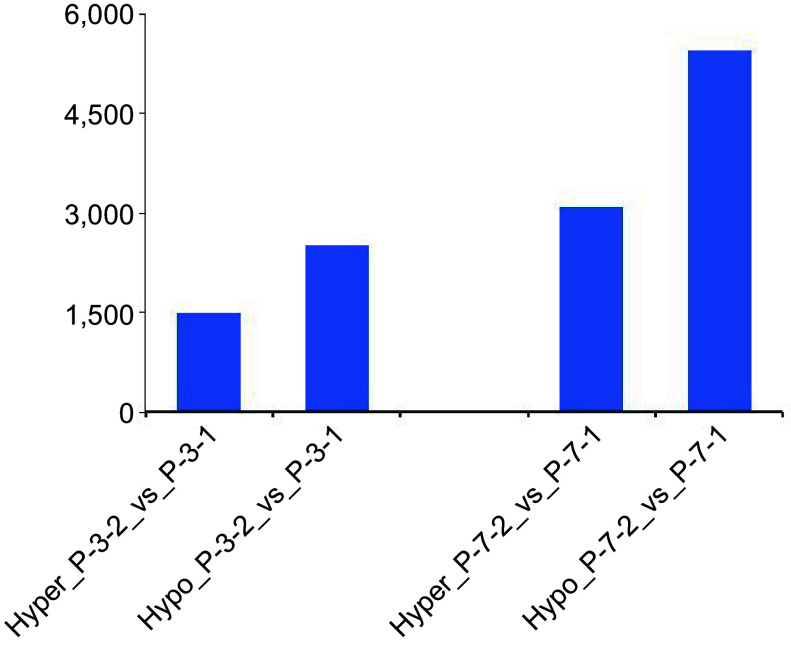
Numbers of hypermethylated/hypomethylated differentially methylated regions between embryogenic tissues (P_3_1, P_7_1) and 5-aza-dC treated embryogenic tissues (P_3_2, P_7_2) during the pre-maturation treatment.

MeDIP sequencing was performed to explore the DNA methylation modifications during 5-aza-dC treatment (Fig. 5). Untreated embryogenic tissues (P_0, P_3_1, P_7_1) and 5-aza-dC treated embryogenic tissues (P_0, P_3_2, P_7_2) were sampled at 0, 3, and 7 d. The number of differentially hypermethylated/hypomethylated regions was 1499 for P_3_2 and 2516 for P_3_1, whereas the number of differentially hypermethylated/hypomethylated regions was 3090 for P_7_2 and 5449 for P_7_1. Particularly noteworthy was the higher number of hypomethylated regions between untreated embryogenic tissues (P_3_1, P_7_1) and 5-aza-dC treated embryogenic tissues (P_3_2, P_7_2), indicating that 5-aza-dC treatment exerts a significant effect on immature SEs.

**Figure 6:**
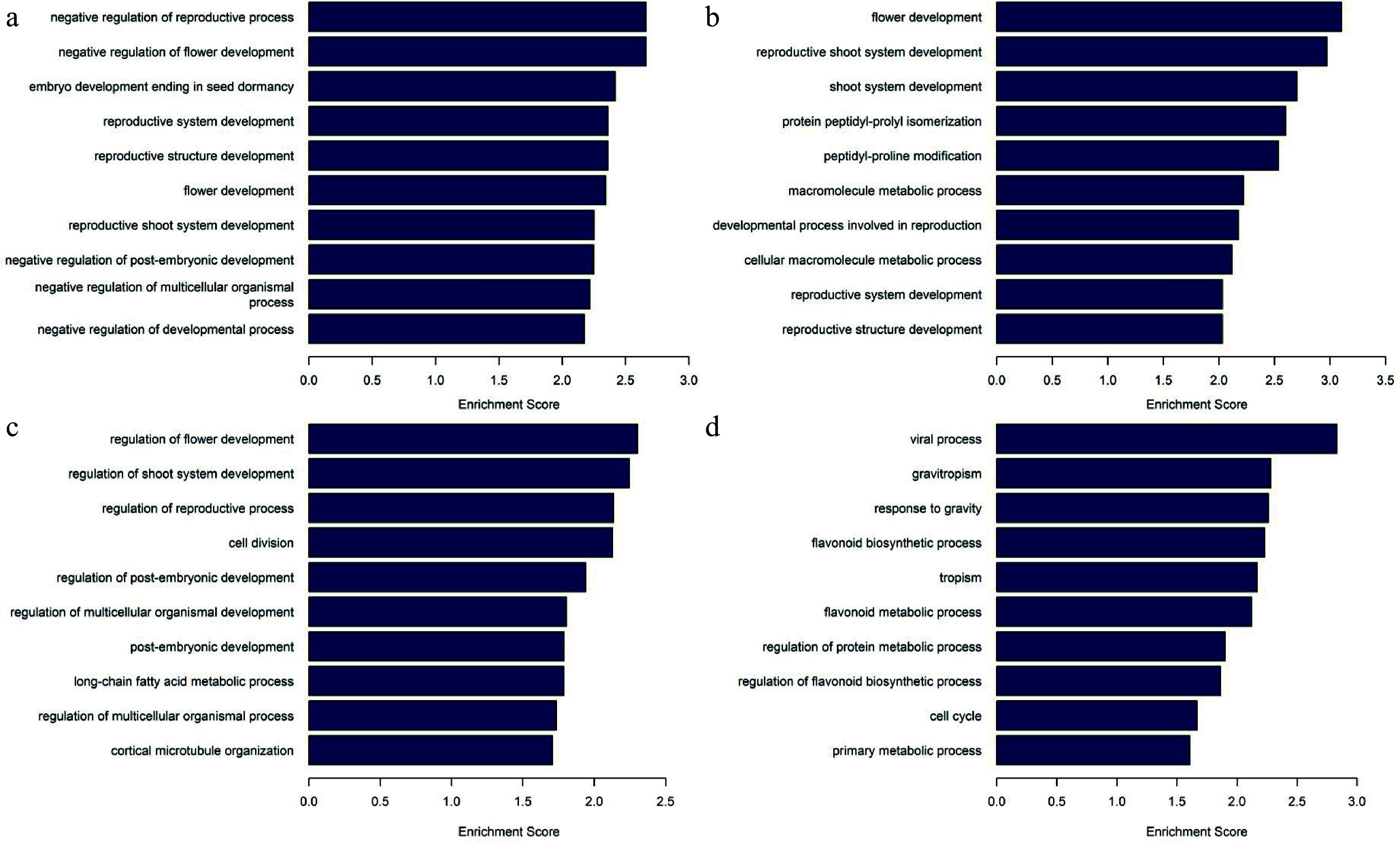
Gene Ontology analysis and biological processes of differentially methylated regions. (a) Enriched biological processes in differentially hypermethylated regions of P_3_2_vs_P_3_1. (b) Enriched biological processes in differentially hypermethylated regions of P_7_2_vs_P_7_1. (c) Enriched biological processes in differentially hypomethylated regions of P_3_2_vs_ P_3_1, (d) Enriched biological processes in differentially hypomethylated regions of P_7_2_vs_ P_7_1.

As for the corresponding genes of the promoter in differentially methylated regions, GO analysis showed that the functions of differentially hypermethylated regions between P_3_2 and P_3_1 were mainly enriched in 'negative regulation of reproductive process', 'negative regulation of flower development', and 'embryo development ending in seed dormancy', whereas differentially hypomethylated regions were mainly enriched in 'regulation of flower development', 'regulation of shoot system development', 'regulation of reproductive process', 'cell division', and 'regulation of post-embryogenic development' (Fig. 6). Comparative analysis of P_7_2 and P_7_1 showed that biological processes such as 'flower development', 'reproductive shoot system development', 'shoot system development', 'protein peptidyl-prolyl isomerization', and 'peptidyl-proline modification' were enriched for differentially hypermethylated regions, whereas 'gravitropism', 'response to gravity', 'flavonoid biosynthetic process', and 'tropism' were enriched for differentially hypomethylated regions. In contrast to the results on the 3^rd^ day, genes related to development and reproduction were hypermethylated, whereas genes related to polarity and secondary metabolites were hypomethylated, suggesting that embryo development is inhibited after 7 d of 5-aza-dC treatment.

### RNA sequencing and differential analysis of embryogenic and epigenetic-related genes

**Figure 7:**
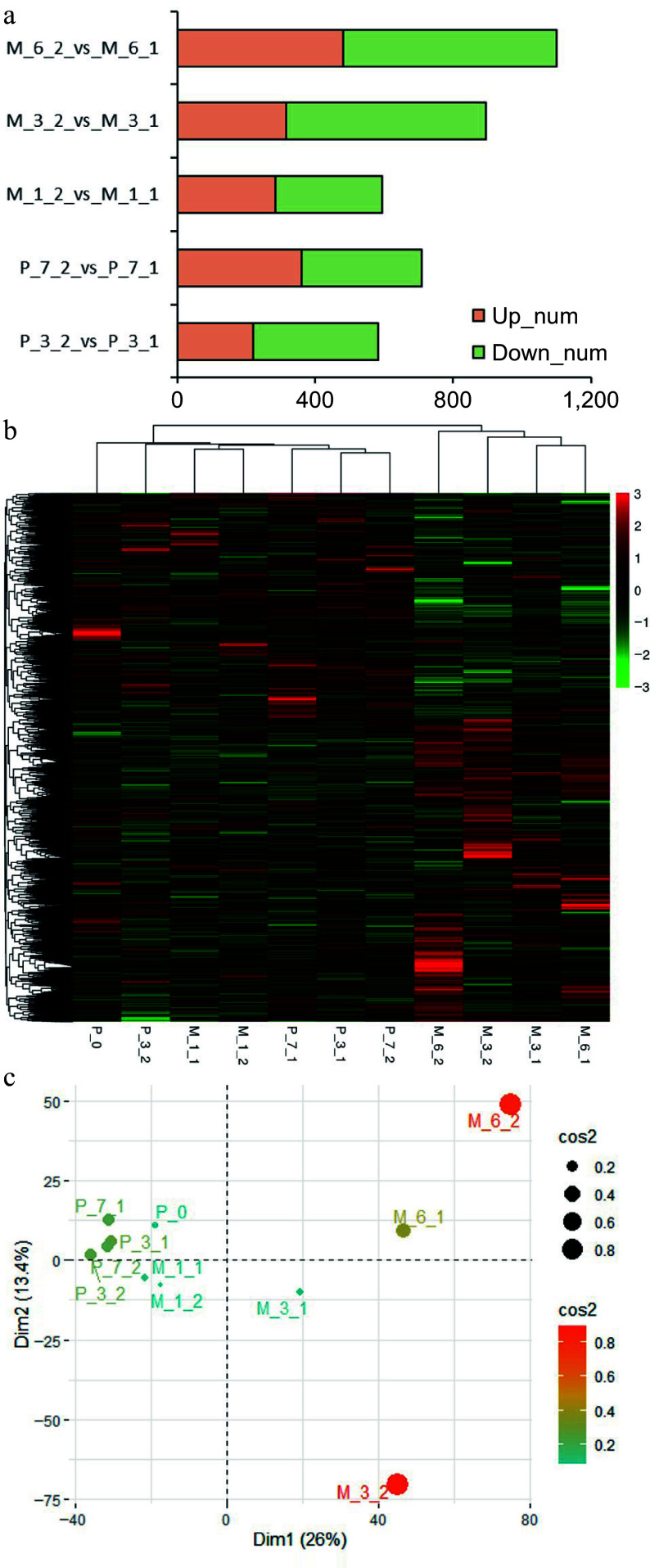
Results of RNA sequencing. (a) The differentially expressed genes between untreated (P_3_1, P_7_1, M_1_1, M_3_1, M_6_1) and 5-aza-dC treated (P_3_2, P_7_2, M_1_2, M_3_2, M_6_2) embryogenic tissues on the 3^rd^ day (P_3) and 7^th^ day (P_7) of pre-maturation and the 1^st^ week (M_1), 3^rd^ week (M_3), and 6^th^ week (M_6) of maturation. (b) Heatmap of clustering analysis with all samples (P_0, P_3_1, P_7_1, P_3_2, P_7_2, M_1_1, M_1_2, M_3_1, M_3_2, M_6_1, M_6_2). (c) PCA analysis with all samples.

To explore the impact of DNA methylation modifications on gene expression, RNA-seq was performed using samples from untreated and 5-aza-dC treated embryogenic tissues during pre-maturation at 0, 3, and 7 d (P_0, P_3, P_7) and maturation at 1, 3, and 6 weeks (M_1, M_3, M_6). A total of 5970 genes were obtained with |(fold change)| > 5. Comparing 5-aza-dC treated embryogenic tissues (P_3_2, P_7_2, M_1_2, M_3_2, M_6_2) with untreated embryogenic tissues (P_3_1, P_7_1, M_1_1, M_3_1, M_6_1), the number of differentially expressed genes increased with the development of SEs, reaching 1099 genes by the 6^th^ week. Notably, the number of differentially downregulated genes between 5-aza-dC treated and untreated embryogenic tissues was similar at early developmental stages but sharply increased to 581 genes by the 3^rd^ week. Meantime, clustering and PCA analysis (Fig. 7) showed that genes at early stages (P_0, P_3_1, P_7_1, P_3_2, P_7_2, M_1_1, M_1_2) exhibited similar expression trends, which was different from genes at late stages (M_3_1, M_3_2, M_6_1, M_6_2), indicating that 5-aza-dC treatment leads to an increase in downregulated genes which impedes somatic embryogenesis.

**Figure 8:**
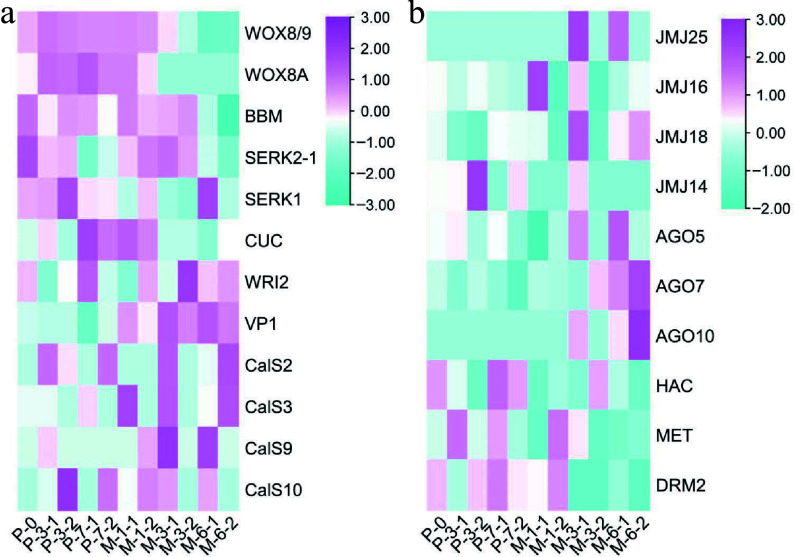
Results of heatmap. (a) The expression profile of embryogenic genes during the development of SEs. (b) The expression profile of epigenetic genes during the development of SEs.

To further explore the impact of 5-aza-dC treatment on gene expression throughout somatic embryogenesis, the expression profiles of embryogenic-related genes (Fig. 8a) and epigenetic-related genes (Fig. 8b) were analyzed. Among the embryogenic-related genes, the expression levels of *WOX8/9* (*WUSCHEL homeobox protein WOX8/9*), *WOX8A* (*WUSCHEL homeobox protein WOX8A*), and *BBM* (*Transcription factor baby boom*) decreased with somatic embryogenesis. The expression levels of *SERK2-1* (*Somatic embryogenesis receptor kinase 2-1*), *SERK1* (*Somatic embryogenesis receptor kinase 1*), and *VP1* (*Transcription factor viviparous 1*) in early untreated embryogenic tissues were higher than those in early 5-aza-dC treated embryogenic tissues but their levels decreased at later stages of somatic embryogenesis. Conversely, *CUC* (*Cup-shaped cotyledon*) and *WRI2* (*Wrinkeled2*) showed an opposite expression trend. Notably, the expression level of *CalS10* (*Callose synthase 10*) was highest in early 5-aza-dC treated embryogenic tissues, decreasing at later stages of somatic embryogenesis, which was different from the other genes of the callose synthase family. For epigenetic-related genes, *JMJ25* (*lysine-specific demethylase JMJ25*), *JMJ16* (*lysine-specific demethylase JMJ16*), *JMJ18* (*lysine-specific demethylase JMJ18*), *AGO5* (*Argonaute protein 5*), *AGO7* (*Argonaute protein 7*), and *AGO10* (*Argonaute protein 10*) were upregulated at late stages of somatic embryogenesis, whereas *JMJ14* (*lysine-specific demethylase JMJ14*), *HAC* (*histone acetyltransferase HAC1*), *MET* (*Methyltransferase*), and *DRM2* (*Domains rearranged methyltransferase 2*) were upregulated at early stages of somatic embryogenesis. Notably, the expression profile of *JMJ14* was different from that of the other JMJ genes, exhibiting higher expression levels in 5-aza-dC treated embryogenic tissues at early stages and lower expression levels at late stages of SE development. These results further illustrate the significant impact of 5-aza-dC treatment on gene expression during the early stages of somatic embryogenesis.

### WGCNA analysis

**Figure 9:**
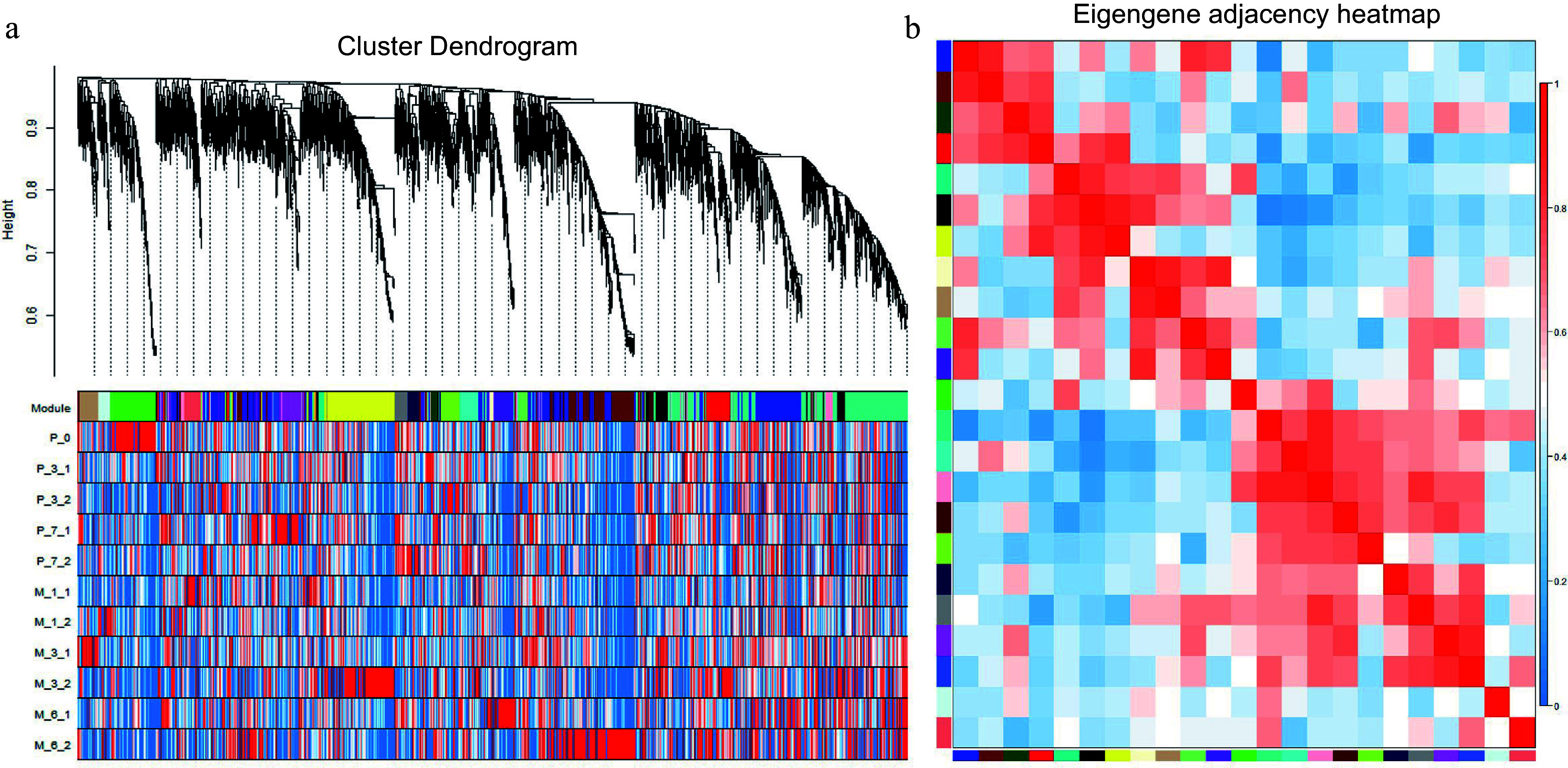
WGCNA analysis of 5970 differentially expressed genes. (a) Hierarchical cluster tree showing coexpression modules with differential expression trends identified by WGCNA. Each leaf in the tree is one gene, and the expression profile of each gene is displayed in the following heatmap. The major tree branches constitute 23 modules labeled by different colors. (b) Eigengene adjacency heatmap. Each row and column correspond to a module.

**Figure 10:**
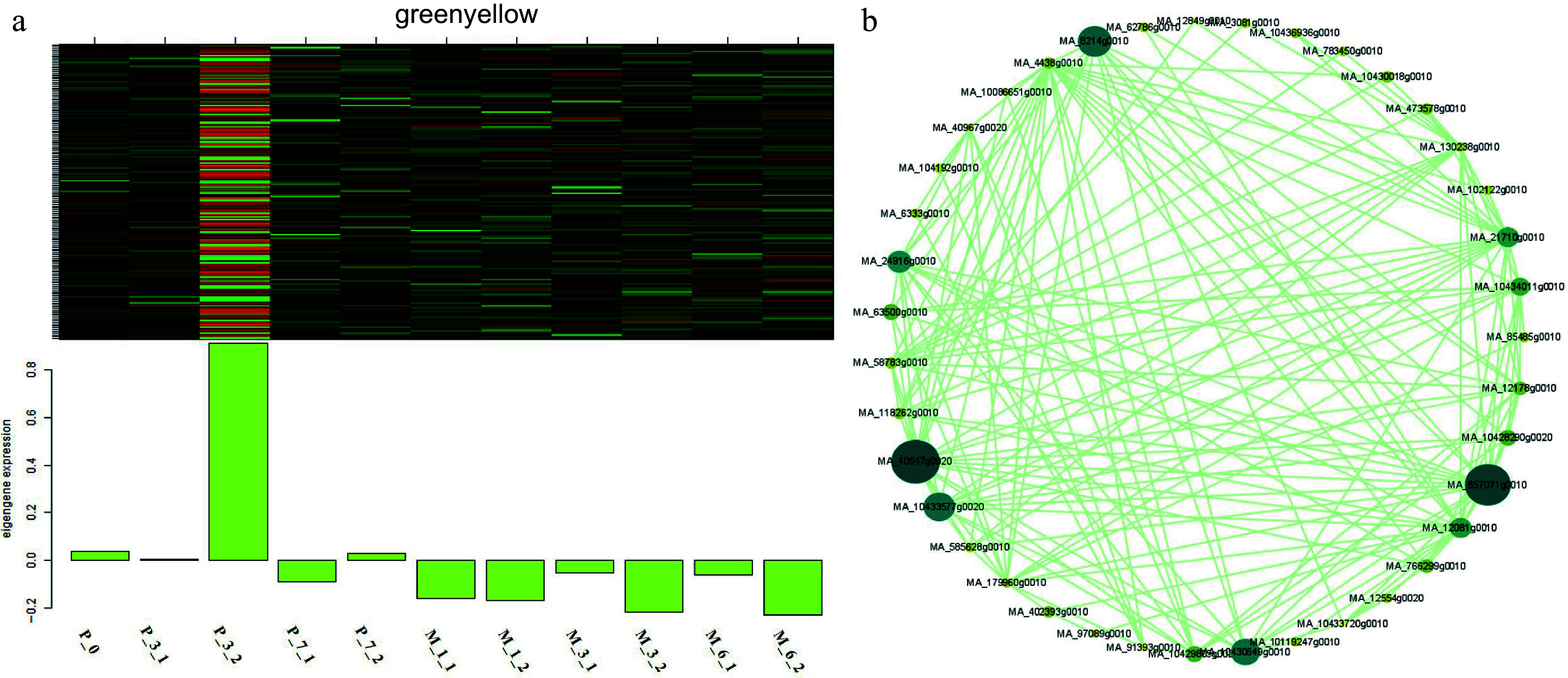
Expression module and correlation network associated with the sample-specific modules. Heatmaps of (a) greenyellow module show the expression profile of all co-expressed genes in the modules (labeled on top). The color scale represents the Z-score. Bar graphs (below the heat maps) show the consensus expression pattern of the co-expressed genes in each module. (b) Correlation network of genes within the greenyellow module.

**Figure 11:**
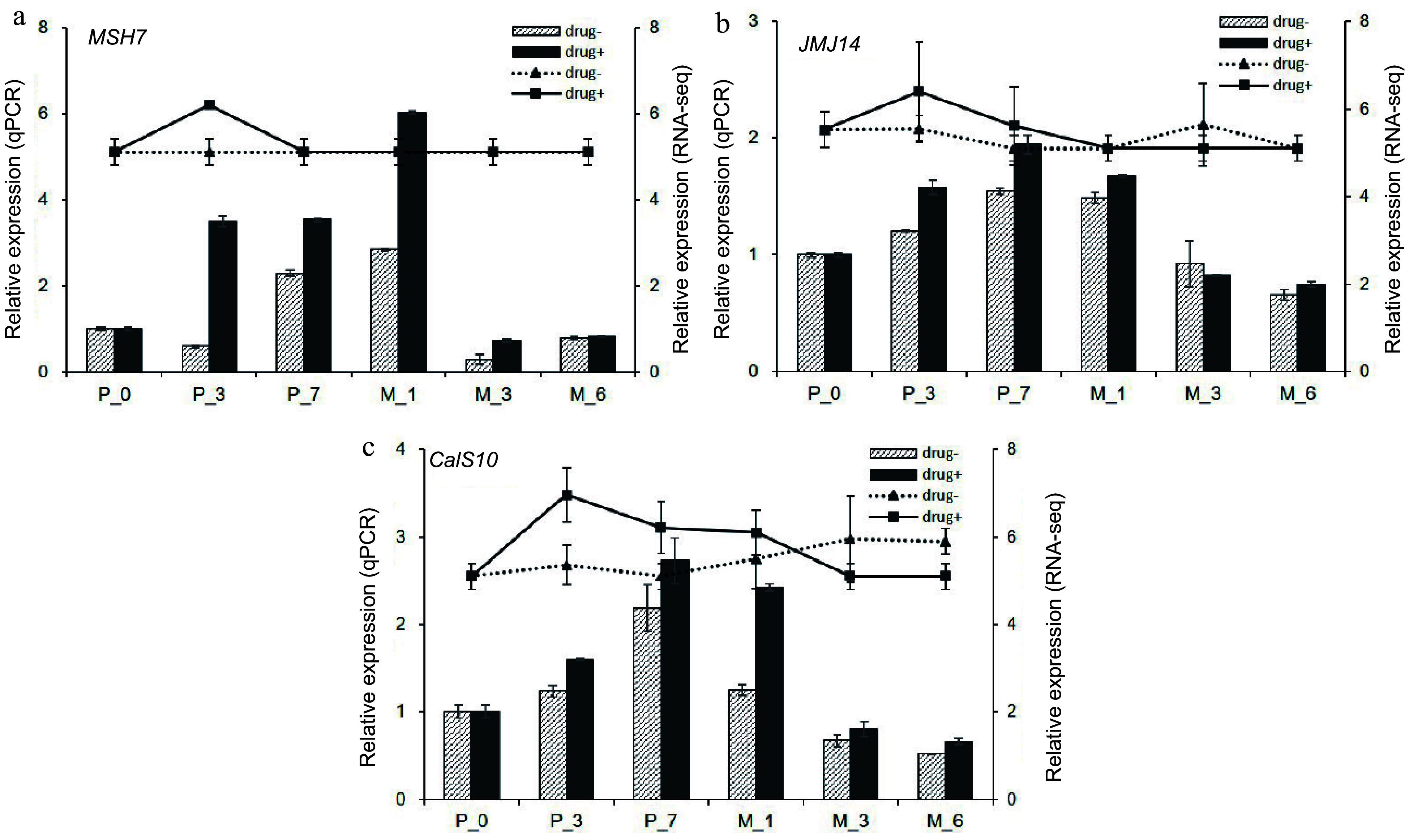
qPCR verification of hub genes. qPCR results of (a) *DNA mismatch repair protein MSH7-like* (*MSH7*), (b) *lysine-specific demethylase JMJ14* (*JMJ14*), and (c) *callose synthase 10* (*Cals10*). Drug+ represents 5-aza-dC treated embryogenic tissues, and drug- represents untreated embryogenic tissues. The columns represent the qPCR expression level related to the left axis, while the lines represent the RNA-seq expression level correlated with the right axis.

WGCNA analysis was carried out to identify key genes involved in somatic embryogenesis (Fig. 9). A total of 5,970 differentially expressed genes in 5-aza-dC treated SEs were classified into 23 modules, and the 'greenyellow' module corresponded to genes that were upregulated at early stages and downregulated at late stages of somatic embryogenesis compared with untreated SEs (Fig. 10). *DNA mismatch repair protein MSH7-like* (MA_40647g0020, *MSH7*) is one example of a gene that was significantly upregulated in 5-aza-dC treated SEs (Fig. 10). Meanwhile, the embryogenic-related gene *callose synthase 10* (MA_12849g0010, *Cals10*) and the epigenetic-related gene *lysine-specific demethylase JMJ14* (MA_97089g0010, *JMJ14*) were also identified and verified through qRT-PCR (Fig. 11). Therefore, 5-aza-dC treatment altered the expression levels of *MSH7*, *Cals10*, and *JMJ14*, suggesting that *MSH7*, *Cals10*, and *JMJ14* may play a critical role in SE development.

### Integrated methylated regions and gene expression analysis

**Figure 12:**
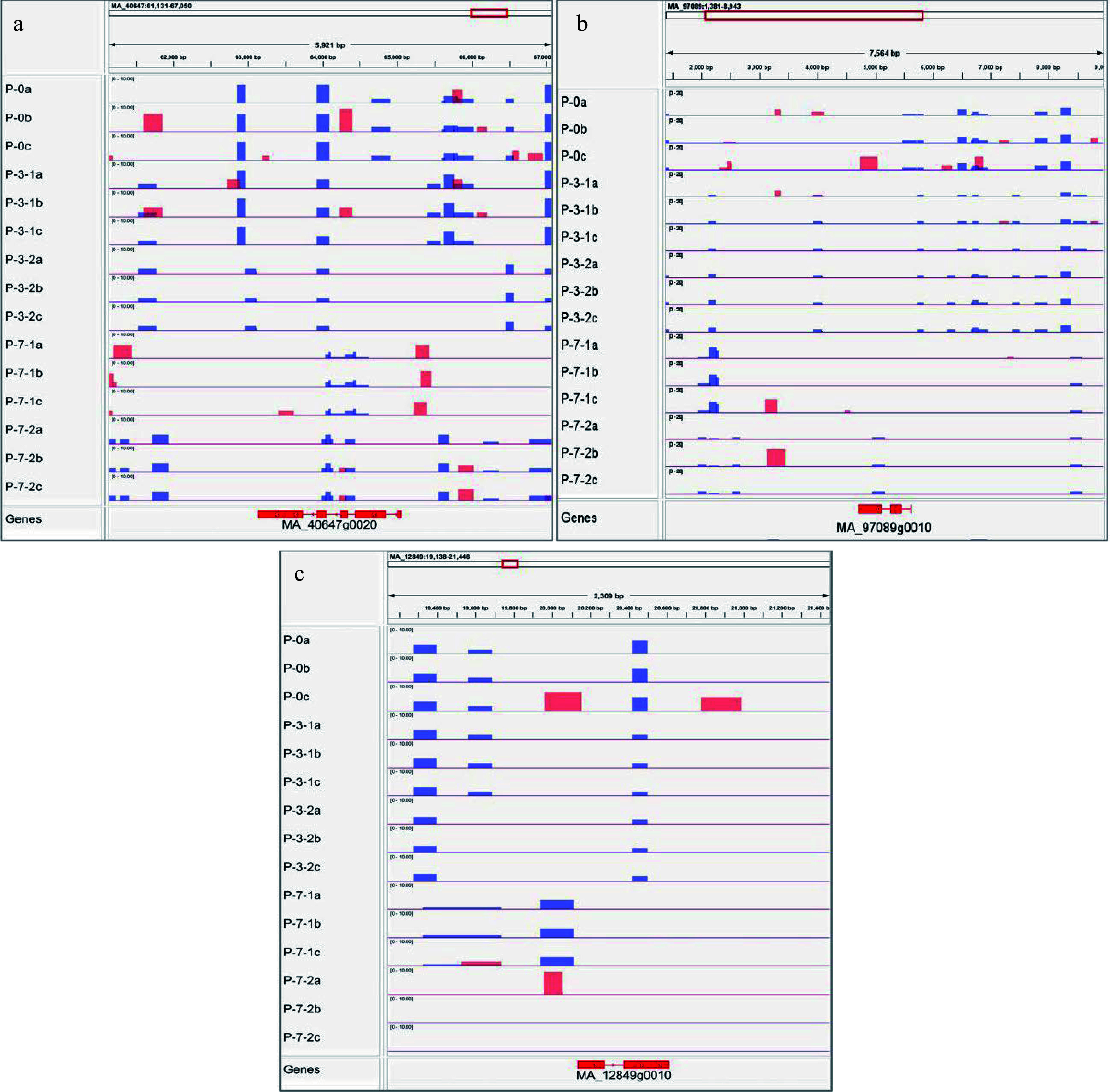
Visualization results of differentially methylated regions. MeDIP results of (a) *DNA mismatch repair protein MSH7-like* (*MSH7*), (b) *lysine-specific demethylase JMJ14* (*JMJ14*), and (c) *callose synthase 10* (*Cals10*). Red peak represents IP, blue peak represents Input.

In this study, the DNA methylation modifications and gene expression trends of *MSH7*, *Cals10*, and *JMJ14* were integrated to further explore the correlation between DNA methylation modifications and gene expression trends. As shown in Fig. 12, the DNA methylation regions and gene expression levels of *MSH7*, *Cals10*, and *JMJ14* were significantly different at various development stages in primary embryogenic tissues. Meanwhile, the expression levels of *MSH7*, *Cals10*, and *JMJ14* were lower in primary embryogenic tissues than those exposed to pre-maturation treatment (Fig. 11). Notably, there were few methylated regions on the 3^rd^ day of 5-aza-dC treatment, and the extent of DNA methylation was lowest during pre-maturation treatment, whereas the expression levels of *MSH7*, *Cals10*, and *JMJ14* were upregulated. These results indicate that the impact of 5-aza-dC treatment is evident on the 3^rd^ day of pre-maturation treatment, at which time it promotes biological processes such as 'regulation of reproductive process', 'cell division', and 'regulation of post-embryonic development'. On the 7^th^ day of pre-maturation treatment, the extent of DNA methylation was slightly increased, unlike that in primary embryogenic tissues, which was correlated with downregulated gene expression. Therefore, the expression and methylated regions of *MSH7*, *Cals10*, and *JMJ14* can be closely linked, suggesting that DNA methylation modifications in *MSH7*, *Cals10*, and *JMJ14* may be involved in somatic embryogenesis.

## Discussion

DNA methylation plays an important role in plant phenotype by modulating gene expression^[34]^. In this study, the demethylation treatment applied to early immature SEs resulted in an increased occurrence of immature and abnormal embryos, thus impeding the further development of SEs. Meanwhile, compared to untreated SEs, the expression levels and methylated regions of specific genes, namely *MSH7*, *Cals10*, and *JMJ14*, exhibited obvious differences in treated SEs. Thus, these findings indicate that SE development is intricately linked to modifications in DNA methylation patterns, and deviations from these patterns can lead to abnormal somatic embryogenesis.

### Cytoxicity of 5-aza-dC

5-aza-dC exhibits low toxicity and a potent effect on DNA demethylation, making it widely employed in research related to embryo development^[35]^, cancer therapy^[36]^, and cell differentiation^[37]^. Currently, 5-aza-dC has found application in studies concerning secondary metabolites^[38]^, seed quality^[39]^ and stress response^[40,41]^ in plants. Additionally, it has been utilized in plant suspension cultures to prevent cells from undergoing methylation^[42,43]^. Therefore, in this study, 5-aza-dC was the optimal choice for treating suspension SEs during the pre-maturation stage.

Notably, 5-aza-dC demonstrated minimal impact on the viability of early SEs at concentrations below 2.0 µM. However, when the concentration of 5-aza-dC reached 2.5 µM, the early SEs of white spruce exhibited damage by the 7^th^ day. In *Cephalotaxus mannii*, the enzyme activity that is associated with cell growth and product biosynthesis markedly improved at the optimal concentration of 5-aza-dC, which was 15 μmol/L^[42]^. Conversely, the viability of triticale microspores decreased with 20 µM 5-aza-dC treatment^[43]^. The results underscore the concentration-dependent effect of 5-aza-dC on the growth of early SEs.

### DNA methylation in somatic embryogenesis

Recently, it has been demonstrated that alterations in the early developmental program of embryos coincide with changes in global DNA methylation. The global DNA methylation level of SEs treated with 5-aza-dC exhibited a significant decrease on the 7^th^ day of pre-maturation and at the 1^st^ week of maturation, resulting in lower maturity rates and higher malformation rates. Munksgaard et al. observed an increase in the concentrations of S-adenosylmethionine (SAM) and S-adenosylhomocysteine (SAH) during the development of carrot SEs, accompanied by a subsequent increase in DNA methylation levels^[44]^. Another study indicated that CHH hypermethylation is both autonomous and conserved throughout embryo development^[45]^. These results suggest the essential and conserved role of DNA methylation in somatic embryogenesis.

Demethylating treatments not only reduce the global DNA methylation level but also alter the distribution locus of the methylated DNA within embryo cells. Despite the increase in global DNA methylation levels up to the 7^th^ day, the number of hypomethylated regions exhibited a sharp increase, while the number of hypermethylated regions increased slightly on the 3^rd^ day of 5-aza-dC treatment. These findings indicate that both global DNA methylation and specific methylation status undergo modifications, and there is no direct correlation between global DNA methylation and specific methylation status.

DNA demethylation enhances the totipotency of embryogenic tissues but hinders the maturation of SEs, as observed in *Coffea canephora*^[46]^ and *Arabidopsis*^[47]^. Similarly, in *P. glauca*, when comparing hypomethylated regions in 5-aza-dC treated embryogenic tissues with untreated ones, the former exhibited enrichment in 'regulation of reproductive process', 'cell division', and 'regulation of post-embryonic development' on the 3^rd^ day. However, by the 7^th^ day, the enrichment shifted to 'gravitropism', 'response to gravity', 'flavonoid biosynthetic process', and 'tropism', indicating that demethylation treatment promotes and preserves the division ability of embryos but also inhibits the development of SEs.

### Integrated DNA methylation and gene expression during somatic embryogenesis

The DNA cytosine methylation pattern plays an essential role in the transcriptional regulation of gene expression, serving as a determining factor in plant growth and development^[48,10]^. Chemically induced DNA modification has the potential to activate or deactivate gene expression, depending on developmental stages and environmental stresses^[49]^. Compared to untreated embryogenic tissues, *JMJ14* showed demethylation and a higher expression level in early stage SEs. The upregulated JMJ proteins, known for removing repressive H3K27me3 marks, facilitate the accessibility of genomic regions, allowing adaptation to environmental changes, developmental stages, and specific cell types^[50]^. Conversely, the removal of *JMJ14* leads to increased DNA methylation in the promoter region, resulting in the attenuation of gene transcription levels^[51,52]^.

In conifers, somatic embryogenesis serves as an excellent system for both vegetative propagation and theoretical research^[5]^. Well-known genes such as *WOX*^[53]^, *BBM*^[54]^, *SERK*^[55]^, *VP1*^[56]^, *CUC*^[57]^ and *WRI2*^[58]^ play critical roles in somatic embryogenesis. In this study, *CalS10* (*callose synthase 10*) was upregulated during the early stages of SE development due to DNA demethylation induced by 5-aza-dC treatment. A previous study has indicated that the callose layer serves as a marker for cells induced to embryogenesis, which is required for SE^[59]^. Furthermore, a positive relationship has been observed between the callose content and the number of globular SEs^[60,61]^. In the case of embryogenic tissues treated with 5-aza-dC, the elevated expression of *CalS10* coincided with the higher number of early SEs, resulting in the reduction of mature SEs. These results indicate that the heightened expression of *CalS10* may be useful for cell division during the early stages of SE development and can impede somatic embryogenesis.

Remarkably, the DNA methylation pattern of *MSH7* shows variations across different plant species^[62]^. *MSH7* is a plant-specific protein associated with the mismatch repair system, responding to various DNA damage inducers such as nucleotide methylation, oxidative DNA damage, and UV-induced DNA damage, and it corrects mispaired or unpaired bases^[63]^. In this study, chemically induced demethylation brought attention to the *MSH7* gene, and the modification of methylated regions of *MSH7* was closely associated with gene expression, indicating a connection between epigenetic mechanisms and *MSH7* expression during the early stages of SE development. Indeed, the expression of various genes is subject to epigenetic regulation, allowing adaptation to various environmental stresses^[64]^. Although the methylated regions of *CalS10* underwent alterations, it remains unclear whether the elevated expression of *CalS10* resulted directly from DNA demethylation or *JMJ14*-mediated regulation. Further investigations are necessary to evaluate whether the DNA methylation status of *JMJ14* plays a role in controlling gene expression during the development of SEs.

## Conclusions

In summary, the impact of 5-aza-dC treatment on somatic embryogenesis varied based on the dosage and application time, leading to either a promotion of cell division and an increase of early immature SE number, or an inhibition of SE development. This study elucidated that the DNA methylation pattern played a pivitol role in regulating the expression of various genes and influencing SE development through 5-aza-dC treatment. Moreover, in this study, the genes *MSH7*, *JMJ14*, and *CalS10* stood out due to their elevated expression levels and noticeable DNA methylation modifications during the early stages of SE development in 5-aza-dC treated embryogenic tissues. Further studies are needed to unravel the interactions among these three genes.

## SUPPLEMENTARY DATA

Supplementary data to this article can be found online.

## Data Availability

The datasets generated during and/or analyzed during the current study are available from the corresponding author on reasonable request.
