# Supplementary material for: Involvement of 5mC DNA demethylation via 5-aza-2'-deoxycytidine in regulating gene expression during early somatic embryo development in white spruce (Picea glauca)
Source: For Res (Fayettev). 2023 Dec 26;3:30. doi: 10.48130/fr-0023-0030 (PMC11543301; doi:10.48130/fr-0023-0030)
Supplement: Supplementary file 1 — Supplementary data to this article can be found online. [file fr-0023-0030-Suppl-TableS1.pdf]

Supplementary Table S1. qPCR primers for genes in *Picea glauca*.

Gene	Reference genome No.	Forward primer (5′-3′)	Reverse primer(5′-3′)
MSH7	MA_40647g0020	TTTTCTGCCGCCCGAGTTAT	TGTGAGCCTGTTTTGTCAGC
JMJ14	MA_97089g0010	GGCCTGAATGACCAAAGGTG	AGCGTAGTCTCGGCAAACAG
CalS10	MA_12849g0010	GGGCATGGGTATTCTCCTGTT	GGCTTGTTTCCCGCAAGAAT

The reference genome is Pabies-1.0 of *Picea abies.*
